# Book Reviews

**Published:** 1811-01

**Authors:** 


### CRITICAL ANALYSIS

OF

### RECENT PUBLICATIONS

IN THE

### DIFFERENT BRANCHES OF PHYSIC, SURGERY, AND MEDICAL PHILOSOPHY,

---

*Transactions of the Medical Society of London.* Vol. I. Part 1. 8vo. pp. 247. 1810. Maxwell.

THE Society has changed the title of its volume, as well as the mode of publishing it. Transactions is certainly a more appropriate term for such a work than Memoirs, and by being favoured with it in parts, at shorter intervals, our curiosity is gratified without our patience being exhausted.

The volume commences auspiciously, with a paper from Mr. Mason Good, on Medical Technology. After eloquently descanting on the imperfection of our language, and the consequent confusion of our ideas, Mr. Good refers the sources of the corruption to the following heads : "First, the intermixture of different tongues that have no family or or dialectic union. Secondly, the want of a common principle in the origin or appropriation of terms. Thirdly, the introduction of a variety of useless synonyms, or the adoption of different words by different writers to express the same idea. Fourthly, imprecision in the use of the same terms. Fifthly, an unnecessary coinage of new terms upon a coinage of new systems." P. 4.—He then makes some remarks upon each of these sources, and concludes "with a few hints for such a general correction and improvement of medical language as may yet be introduced into it without violence or ostentation."—Mr. Good has been very successful in detecting errors and imperfections; he has handled his flail dexterously, and the chaff has flown in all directions. If he has not been equally fortunate in suggesting remedies for all the evils which he has detected, it is, we apprehend, rather from the difficulty of the subject, than from any lack of ingenuity on his part.

We quote the following specimen of his powers in the exposition of erroneous terms, almost at random.

"What is the meaning of tone? In therapeutics, in physiology, and in the common language of mankind, *sound and healthful elasticity:* that voluntary reaction or state of extension between antagonist muscles, as Galen has admirably observed after Hippocrates, by which they are removed from a modification of rest; and in which the one yields to the other, not from utter debility, but in a precise ratio to the superiority of power exercised over it.* Whence that class of medicines which contribute to this elasticity or healthful reaction in irritable or weakened organs is denominated *tonics;* while organs which are destitute of it are said to be in a state of *atony*. But if *tone* be used to imply health, and *tonics* restoratives of health, what are we to understand by the phrase *tonic spasm?* a phrase founded upon an erroneous physiology, perpetually, as I fear, leading us astray in our practice, and applied to a state of muscle in which there is no more *tone*, elasticity, or healthful reaction, than in the frozen strings of a violin. To shew the full force of the absurdity of this phrase, it is only necessary to translate it; and to tell the English reader that it means neither more nor less than *extensible contraction.*

*Phrenitis* is a common and very proper term for phrensy or inflammation of the brain; but *paraphrenitis* is employed to express inflammation of the diaphragm. How is this last sense to be explained? The medical lexicographers tell us that the preposition παρα is here used as a diminutive to denote a kind of sympathetic phrensy. Yet nothing can be more superficial; and here, as in the former case, the term is derived from an erroneous physiology. *Phren* (φρην) in *paraphrenitis*, has a reference to the very vulgar but very early opinion of the residence of the soul in the præcordia; while *para*, instead of diminution or defect, implies proximity—*inflammation around the seat of the soul.* Yet the meaning of παρα is about equally divided between the ideas of proximity and defect in the medical use of the term, and hence the student

* De Mot Muscul. lib. ii.

can derive no precise information from its employment. Thus the *paracme* of a fever means its decline; *paracusis* depraved hearing; *paranœa* defective judgment; in all which παρα is used diminutively: while in *parotis*, *parathenar*, *paronychia*, it implies proximity alone. In paralysis its intention is doubtful, for it may be taken either way. There are many other prepositions and particles which, in composition, are used in the same indeterminate manner, and considerably augment the confusion of our vocabulary." P. 30.

Having happily pointed out the "*extraordinary intertexture*, the *discordia concors*" of the medical language of the day, Mr. Good proceeds to offer his assistance in correcting it. He advises us "to discard all equivocal terms as much as possible; and in cases where this cannot be done, to assign a fixt (fixed) and individual sense to every term, and never o employ it in any other sense." He recommends us to create as few new words as possible; and among those already in use, to confine ourselves to the same term to express the same idea, even where we have a choice of numerous synonyms. He wishes us "to limit our nomenclature as much as possible to one language alone;" and prefers the Greek, because "by far the greater part of our technology is already derived from it, and that it possesses a facility of combination to which the Latin has no pretensions." We are then, "to banish every Latin, as well as well as every Arabic, Spanish, Italian, and German word in favour of its Greek synonym." But as many diseases, such as *syphilis*, *small-pox*, &c. were unknown to the Greeks, we must condescend to explore the writings of more modern authors to enrich our vocabulary.—Mr. Good concludes with submitting the following regulations:

"1 Let the particle a (α) express alone the idea of total privation; as in *amentia*, *agalactia*, *amenorrhœa*.

"2. Let dys (δυς) express alone the idea of deficiency, as its origin δυνω or δυμι most naturally imports, and as we find it employed to express in *dys-pnœa*, *dys-cinesia*, and *dysphagia*.

"3. As an opposite to dys, let *en* (εν) be employed as an augmentive particle, as we have it in *en-harmonic*, *en-telechia*, and *en-ergetic*. *En* is not often, indeed, a medical compound, nor do I recollect its being employed in more than two instances; *encephalon*, in which it has the sense of *interior* (a word, indeed, that has been long falling into disuse), and *enuresis*, in which it imports excess, and is consequently used as now recommended. Thus restricted, εν and δυς will have the force of υπερ and κατω, but will be far more manageable in the formation of compounds.

"4. Let *agra* (αγρα) be restrained to express the idea of simple morbid affection in an organ, synonymously with the Latin *passio*, or the *berh* of the Arabians.

"5. Let *itis* (ιτις) express alone the idea of inflammatory action, as in *cephalitis*, *gastritis*, *nephritis*.

" 6. Let *algia* (αλγια) express alone the idea of pain or ache, to the banishment of such useless synonyms as *odyne* and *copos* or *copus*.

" 7. Let *rhagia* (from ρησσω *rumpo*) be confined to express a præternatural flux of blood.

" 8. Let *rhæa* (from ρεω *fluo*) express a præternatural flux of any other kind."

A table of radical compounds is subjoined.

We give the author credit for the learning and the ingenuity which he has displayed in this essay, but we have no apprehension of being obliged by his verbal speculations, to disburthen our memory of the "*gibberish*" acquired from ancient writers, and charge it with new terms, which have not always the merit of being more appropriate, and in many instances, are more barbarous and cacophonous. Thus we have *enenteria* for diarrhæa; *gastrorrhagia* for hæmatemesis; *pneumonorrhagia* for hæmoptoe; *urirrhagia* for hæmaturia; *urirrhœa* for pyuria; *ophthalmalgia* for ache of the eye-ball. We know that Mr. Good is very conversant with dictionaries, and were therefore surprised at his remarks on the particle *en*, which is used as a compound much more frequently than he seems to be aware of, as in enæorema, enarthrosis, encanthis, encœlia, encauma, endemic, encope, &c. &c. Without any pretensions to the art of prophesying, we may venture to predict, that whilst the works of Hippocrates, of Sauvages, of Cullen, and of numerous other accurate describers of diseases, continue to be read and admired, the terminology which they have used will be adopted. The reform of language is slow and gradual; and till we have better books, and more complete systems of medicine, we must endure the jargon which prevailed in the times when our best treatises were composed; let us bear in mind the opinion of Vaugelas, "Lorsqu'une façon de parler est usitée des bons auteurs, il ne faut pas s'amuser à en faire l'anatomie, ni à pointiller dessus, comme font une infinité de gens; mais il faut se laisser emporter au torrent, et parler comme les autres, sans daigner ècouter ces eplucheurs de phrases."

In the second article, the President, Dr. Lettsom, has drawn up a neat account of the late William Hewson, F. R. & M. S. and Teacher of Anatomy in London. He was born in 1739; and in 1760, took charge of the dissecting room, in the absence of John Hunter, with whom, in 1762, he acted as joint lecturer. In 1768, he was engaged in investigating the anatomy of fishes, and in the following year, presented to the Royal Society, of which he soon afterwards became a member, an account of the lymphatic system in birds and fishes, and particularly in the turtle. In 1771, he published his "Experimental Inquiry into the Properties of the Blood." In the autumn of the following year, the lecturing connection between him and Mr. Hunter being dissolved, he commenced on his own account, and opened his course with a lecture on the uses of the spleen and thymus. In 1774, he published his work on the lymphatic system, and in the spring of the same year, whilst blessed with domestic felicity, and favoured by extensive practice, and well earned celebrity, "he was attacked with a fever, occasioned by a wound which he received in dissecting a morbid body;" and died on the 1st of May, at the age of 35.

Mr. Hewson's celebrity chiefly hinged on his discovery of the lymphatic system in birds and amphibious animals, and his experiments on the blood. Dr. Monro has disputed (we believe unjustly) his claim to the first, and modern chemistry has, in a great degree, destroyed the value of the latter. We must admire his ingenuity and his industry, and lament that the fame of man rests on frail materials.

The third article in the Transactions, contains a "History of fatal effects from the accidental use of white lead;" by John Deering, Surgeon, with additional remarks, by William Shearman, M. D. We shall state the substance of the case as related by Mr. D.

Mrs. R October 21, 1808, "complained of violent pain in the scrobiculus cordis, with great soreness of the epigastric region when pressed upon. She had vomited a considerable quantity of bilious matter, and at the same time her bowels were constipated: the pulse was calm and regular, the tongue clean and moist, and there was no symptom of fever present. She immediately took a cathartic, which operated, and an opiate in the evening." In the morning she was relieved; but in the evening the pains and vomiting returned, and continued for some days very distressing.

Nov. 4. A physician was called in; he considered the affection as rheumatic and spasmodic, and discontinued his visits in a few days, in consequence of the amendment of the patient. "In about a week after this period, a boy in the same family, nearly sixteen years of age, was seized with symptoms exactly similar to those of the preceding case, and similar remedies afforded only partial relief, till at length he was removed into the country, and thereby recovered his health." Another child, soon afterwards, was seized with similar symptoms, the mother relapsed into her former state, and three other persons in the family laboured under analogous affections. Poison was now suspected, but no indication of it, after minute investigation, appeared. The child, in about a fortnight, was pronounced by his physicians in a convalescent state; but was soon after seized with convulsions, and expired within a few hours. Mrs. R. in whom the sickness and pain had continued unabated, now gradually grew a little better "She had hitherto continued to suckle her child, which, it being fifteen months old, she was advised to wean: to this she reluctantly consented. In about ten days afterwards the child became somewhat costive, without any other apparent indisindisposition; but at this period it was seized with vomiting and convulsions, and suddenly expired." The unfortunate mother, after experiencing some relief, without being entirely free from complaint, on the 21st of January, 1809, was seized in the morning with convulsions, which continued till 5 o'clock, P. M. when she expired. On the subsequent day, Mr. Chevalier examined the body by dissection; he could not, however, discover the least trace of morbid affection.

Of the three other persons who were indisposed, two recovered, and one died after lingering under disease till March.

A Committee of the Medical Society proceeded to inquire into this extraordinary case; but no probable cause for the calamity was ascertained, till Dr. Laird detected a white powder adhering to the inner surface of a cask which had contained sugar used by the family. Upon subjecting this powder to heat by means of the blow-pipe, globules of lead in the metallic state were produced. The mystery was now developed. The sugar had been put into a cask which had previously contained white lead, and becoming impregnated with the metal, was, doubtless the source of the fatal events described

"Of nine persons in this family, who were more or less indisposed, four died, and the effects of the poison appear to have been nearly in the ratio of their respective ages."

Dr. Shearman describes an affection of the bowels very similar to the above, which prevailed in a provincial town. The cause of the complaint was traced by Dr. S. to some Holland's geneva, which he found contained a metallic poison. The spirit had been bought at the Excise office, and the chief officer, on being examined before a magistrate, "confessed that the whole of the quantity of Holland's sold at the last sale had been impregnated with sugar of lead, for the purpose of depriving the spirit of the colour which it always contained by being kept some time in the tubs in which it was brought over sea by the smugglers."

Dr. Dixon, of Whitehaven, in the fourth article, relates the history of a case resembling hydrophobia, from the bite of a cat. The animal was not supposed to be mad, but the symptoms of the patient were unquestionably those of a person affected with rabies canina, and terminated in the usual fatal manner. Our limits will not admit of the whole of this interesting case being inserted, and its value would suffer from being abridged or partially detailed.

The fifth article, by Dr. Falconer, of Bath, treats of the "indiscriminate use of mercurial preparations." The author commences with some well expressed animadversions on quackery, a subject indeed, on which little can now be said which has not been advanced by a hundred writers before; of this part of his essay we quote the following with approbation:

"The absurdity of implicit faith in the decision of beings of our own nature and rank in the line of creation, is not confined to religion. Medicine dicine affords numerous instances equally striking, and both perhaps produced by nearly the same cause. The miracles of the Romish church, and the instances of cure produced by empirics, bear a strong resemblance to each other; and the practitioners of medicine in this branch have not much fallen short of their religious associates in the extraordinary instances of success which they exhibit." Pag. 99.

After stating the usual effects of mercury on the system, the learned author proceeds with his inquiry into the operation of calomel in some of the disorders in which it is prescribed. He thinks it is prejudicial in scrofula, and in glandular swellings, being more likely from its "*stimulant and inflammatory* qualities" to lay the foundation of obstruction than to remove it.

"A state of indisposition, marked by a pale leaden-coloured countenance, defect of appetite, paucity of urine, and sense of weight rather than of pain in the abdomen, accompanied with low spirits; a pulse sometimes rather slower than ordinary, but generally irregular in this respect; and often a dry but harsh or corrugated skin, and a degree of animal heat, rather below than above the natural standard; frequently occurs among those who resort to the Bath waters for relief.

"This disease is generally ascribed, how truly I cannot say, to mesenteric obstruction; and the hardness of the abdomen, which sometimes accompanies the other symptoms, seems to countenance this opinion. But this hardness is often very variable, particularly in women, and though at times evident, is at others scarcely perceivable. Such cases are sometimes relieved by the use of the Bath waters, especially in female subjects, but I fear the failures are pretty numerous. These instances however, afford an ample field for the trial of mercurial preparations, especially calomel, which in these, as in most other obscure chronic complaints, where no specific indication is suggested, is usually plentifully administered; in a good measure, it must be owned, on an empirical footing. The symptoms above described, afford but too much reason to suspect scirrhus of some of the viscera, and the ill effects of mercurials in all cases that partake of a cancerous nature are but too well known. If scirrhi of this kind are but of small extent, and indolent, they may often by a cool regimen, a milk diet, some assistance afforded to the general health, and a quiet manner of life, by avoiding extremes of temperature and other causes of irritation, be kept from spreading, and contributing much either to embitter or to shorten life. But the administration of mercurials, of all which, calomel may in the present age be regarded as the representative, is too apt to rouse the slumbering malady into an active inflammatory state, from the consequences of which few escape, and those few, only by such efforts of nature as we have no right to expect, and know not how to promote or to imitate."

In cases of decided hepatitis, Dr. Falconer affirms, the bad effects of calomel are still more evident; in confirmation of which, he relates the case of a gentleman affected with that complaint, in which calomel seemed to aggravate the severity, and to accelerate the fatal termination. He sees no reason reason to prefer mercurial purgatives in simple obstruction of the gall ducts, which often occur without any diesase whatsoever of the liver itself. "Calomel has no specific power in dislodging a biliary calculus." He deprecates the use of calomel in complaints which are often very improperly termed bilious.

"Complaints (he observes) of an obscure nature are denominated bilious, on a strange and even contradictory supposition that they proceed from either a deficiency or redundancy of bile, or from its depraved or corrupted state; though no marks of any of these faults appear, either in the colour of the skin, or the colour, quantity, or rather qualities of the evacuations. It would have been fortunate for mankind, if the practitioners of medicine had done in the present age as Dr. Swift satirically describes them to have done in his time, and, for the cure of these imaginary diseases, to have invented imaginary remedies. But, unfortunately, giants have been brought on the stage to combat pigmies, or rather shadows. Every dabbler in medicine prescribes calomel as freely, and on the most trifling occasions, as he would the most insignificant article in his shop; and this active, and in many instances dangerous, article is smployed oftener, I believe, to the destruction than to the preservation of mankind."

We might quote two pages more on the deleterious effects of this powerful mineral, but we believe they would be familiar to most of our readers. Dr. Falconer has unburdened his conscience in relating them, and we have pleasure in assuring him, that we *read* of many more shocking cases, than we have ever experienced in a very free and extensive use of the remedy, or have observed in the practice of two of the largest hospitals in Britain: Dr. Falconer has himself witnessed some of the evils which he describes, and we cannot doubt his testimony; but it seems to us almost inconceivable, that any intelligent practitioner in the present day can push a remedy beyond due bounds, or persevere in its use, when the direful consequences are manifest. We commend his concluding caution, though we think the practitioner who stands in need of it must be very young.

"I cannot quit this subject without remonstrating in the strongest manner against the too frequent practice of administering on common occasions calomel to young children. It has been found, when largely given, to weaken, and even to disorder, the mental as well as corporeal faculties of grown persons, and the use of it at an early age, when the faculties of either body or mind have not acquired strength and firmness, may both impair the bodily health and debilitate the mental energy of persons, who might, but for such imprudent interference, have distinguished themselves when further advanced in life."

[*To be continued.*]

*Observations on the Climate, Manners, and Amusements of Malta; principally intended for the Information of Invalids repairing to that Island for the Recovery of Health.* By William Domeier, M.D. of the Royal College of Physicians of London, &c. 8vo. pp. 116. Callow. 1810.

The author has divided this little work into four, or rather into five chapters, for there are two chapters IV. The first treats "of the climate and voyage." Malta lies in 35°. of north latitude; the climate is mild and dry, and the atmosphere clear. The thermometer throughout the year is very regular and not subject to sudden changes. In summer it ranges from 70° to 88°; and in winter from 57 to 60°. Dr. Domeier thinks that "this equality must be a great advantage for the recovery and the prevention of those numerous diseases, which are brought on, and maintained by suppression of the cutaneous perspiration, viz. chronic dysentery, diarrhœa, rheumatism, gout, coughs, catarrhs, ophthalmia, cholic, dropsy, cutaneous eruptions, &c." "During three or four months in the summer, it does not rain at all, and rarely even in the winter." "Showers of hail fall once or twice in the winter, but snow never." The dews and fogs are inconsiderable. In September the sirocco (south-east wind) prevails, and renders the air dense and damp. "Persons with flat narrow chests, or those who have diseases in their lungs, such as schirri, vomicæ, ulcers, water in the breast, &c. feel uneasy on the days when the wind blows." The island is free from endemic diseases, and the only epidemic which Dr. Domeier observed during three years residence, was the small-pox, which, however, was much checked by a general inoculation of the cow-pox.

We are informed that a packet leaves Falmouth every three weeks, and arrives at Malta in about a month. The best time for sailing is the middle of August. Consumptive, dropsical, and rheumatic patients, often experience much benefit from the voyage.—The next chapter contains an account "*Of Medical Assistance and Diet*," from which we learn, that the latter is much superior to the former. Provisions of all kinds are excellent, cheap and plentiful, whereas the physicians, except three, are stated to be "without knowledge and judgment," and of the three the best is merely said to understand 'a little of both the English and French languages, to be "acquainted with literature, and a friend of natural history." The second in rank, "cares little for practice, and speaks no English, and only very broken French." The third is a venerable, good man, and one of the the physicians to the hospital. Pharmacy is in a low state, and surgery still worse. The author " wished once to have a cancerous breast of a female patient taken out, but could find no Maltese surgeon who was able to undertake the operation." " I have been present (says Dr. D. speaking of the hospital at Valetta) at the surgical visit after two o'clock in the afternoon in summer, when the surgeon was obliged to dress the patients by candle light. He saw the only window of the end of the ward open, and ordered it to be immediately shut, adding, *that he was not a friend to fresh air for surgical patients.*"

From the two preceding Chapters we learn, that amusement and social pleasures are enjoyed in as great perfection in Malta as at any of our fashionable watering-places in this country, but great temperance and moderation are preserved by all ranks of people in the island. The botanical garden instituted by the late governor Sir A. Ball, affords the inhabitants of " all ages, sexes, and classes," a convenient and pleasant promenade, where " devout monks walk near dashing officers; young, blooming girls wishing to attract notice near decrepid men, who feel no attachment for their neighbour, or for this life altogether; nurses paying more attention to the society than to the infants trusted to their arms; English ladies, tastefully dressed, next Maltese ones, covered with the saldarra*; and to fashionables it is Bond-street." Of course, among this motley groupe we hear of no students.

In the last Chapter we are informed that there are some tolerably good schools in Malta, and a university at Valetta. Of this we are sorry to remark, the medical faculty is extremely defective; perhaps on this account, the inhabitants are prohibited from resorting to the universities of Italy for obtaining instruction, which they cannot receive at home. Amongst the defects in the medical department may be enumerated the appointment of only one lecturer, who is expected to teach all the branches of the healing art in two hours each day. Natural history and experimental philosophy are not taught there; no anatomical theatre, and no clinical lectures are delivered.

The Maltese hospital is attended by four physicians, and four surgeons. These " change every month in performing their duty, so that a patient, who comes in the last day of the month, falls the first of the next immediately under the hands of another practitioner." And " in most cases the whole plan of cure is altered." The medical officers " en-

* A black silken cloak.

deavour to acquire practice by contradicting and blaming one another, and acting otherwise then their colleagues, though not better"—" Besides these mentioned four physicians and surgeons, there is an equal number of under-physicians and under-surgeons employed; also four governors, sub-governors, apothecaries, four chaplains, dressers, persons who only bleed and cup, even one person, who carries smelling bottles at the medical visit, for fear that any body might faint away, (and really the atmosphere is, in some wards, in such a state, that the fear is not ill founded) a person who keeps the linen under his care, even disciples, &c. &c. every one of them is paid, though many are of no use." P. 112. We have now stated the most interesting particulars of this publication; if from the extracts which we have given, our readers should conclude that the author does not consider perspicuity and accuracy essential in composition, we may perhaps avert their censure, by stating that he is a Foreigner.

---

*A familiar Treatise on Asthma, Difficulty of Breathing, Wheezing, and Winter Cough, containing explicit Directions for the Use of the Stramonium Herb.* By James T. Fisher, Surgeon. 8vo. Lond. 1810.

It is certain that facts have arisen fully proving the efficacy of the *Datura Stramonium* in quieting the paroxysm of asthma. In some cases, however, of this disease, it has been inert; in others, it has been injurious. It remains to be pointed out in which it is serviceable, in which inert, and in which injurious. We mean at some future period to collect the evidence for and against this narcotic; and in expectation of gaining information on the subject, we looked into Mr. Fisher's Pamphlet. Though we do not perceive that, in any degree, it elucidates the properties and effects of this species of *Datura*, we fully understand its object in directing where to find a nostrum, a very odd thing, a *Tobacco* made of *Stramonium*. The only way in which it differs from other *empirical ephemera*, is in its abuse of two gentlemen of most respectable characters, and whose talents are an honor to the profession of medicine.

On

*On the Morbid Sensibility of the Eye, commonly called Weakness of Sight.* By JOHN STEVENSON, *Member of the Royal College of Surgeons, &c.* 8vo. London, 1810. p. 108. Highley.

EVERY practitioner has heard more than he has seen of a disease of the eye, under the vague appellation "*weakness of sight.*" This complaint being often esteemed, in certain stages of its progress, rather an inconvenience than an actual disease, has, too generally, been treated with some domestic remedy, or with advertised nostrums. It is no question, but the profession, and society at large, are greatly indebted to those authors who elucidate what have been called minor complaints, with that degree of scientific clearness which makes them rationally understood. The dextrous operator, the describer of unusual occurrences, and the investigator of irremediable calamities, have had, in great abundance, their historians and their eulogists. Mr. Stevenson has sought, and we doubt not, will find reputation in a less brilliant career. In proportion to the frequency of the disease on which he treats, in proportion to the obscurity in which it has hitherto remained, and in ratio agreeing with the precision with which he describes its phenomena, and points out the mode of treatment, will be his claims on public gratitude.

THE complaint which makes the subject of this pamphlet, Mr. Stevenson considers as a "*Morbid sensibility of the Eye*," and that it has not hitherto been described by writers as a distinct disease, and only incidentally noticed: that its remote and proximate causes have been misunderstood, and its cure unfounded on a rational pathology. In endeavouring to give our readers a distinct view of this disease, its causes, and its treatment as stated in this pamphlet, we shall, in a degree, deviate from the arrangement of Mr. Stevenson. According to our idea of perspicuity it will be proper to give a description of the disease, with its usual symptoms; explain its predisponent, exciting, and proximate causes; and conclude with stating the method of cure.

1st. *Description of the Disease.*

"By simply inspecting the eye, it is scarcely possible to recognize the existence of this complaint, as there is not the slightest external ophthalmia; or unusual fullness in the vessels of the conjunctiva, any apparent affection of the biliary glands, nor the least visible organic derangement. The characteristic symptoms are, a morbid sensibility of the eye to light, and different kinds of external stimuli. According to the accurate observations of the late venerable Dr. Heberden, "Oculi si vel levissime sint imbecilles, quamvis nullam morbi notam præ se ferant, agre patiuntur ventum, ignem, pulverem, aut lectionem. *Comment. de morbis oculorum.*) A strong glare of light is always painfully distressing to the patient; and hence, in aggravated cases of this disease, the effulgence of the Sun's rays when admitted to the Eye, excites in it a very acute sensation, which is accurately referred to the bottom of the orbit; around which there is at the same time, a sense of tension and oppressive uneasiness. For the same reason, the patient is miserably uncomfortable in a brilliantly illuminated apartment. In order therefore to exclude the strong and direct rays of light, he instinctively depresses his eyebrows, or applies his hand to his forehead, viewing objects with the palpebræ half closed, by which he is apt to acquire the habit of *blinking. If he attempt to read, or look at small or bright objects, he is soon dazzled, and his vision becomes confused, which added to the pain the effort occasions, speedily compels him to desist. The iris acts with great energy on the admission of the rays of light to the retina, and in consequence, the pupil becomes contracted to a very small aperture; a *striking feature* of this disease. When the stimulus of light affects the Eye, there is sometimes, though very rarely, a manifestly deficient action of the lachrymal glands, but much oftener the secretion of tears is abundantly copious,† which is indeed the principal cause of the confused vision occasionally attending this malady.

2d. *Remote or predisposing Cause.*

"General debility, however, induced, though not essential, seems greatly to predispose to this complaint. Hence, its most frequent occurrence to persons recovering from previous illness, and to those of a relaxed habit. Females are most obnoxious to its attacks, as the predisponent causes are most liable to arise in the low tone of their system.

"Most of the instances of weakness of sight, occur under great constitutional delicacy.

3d. *Exciting Cause.*

"Long or frequent exposure of the eye to a very vivid or reflected light, or its excessive exertion in reading, or viewing minute and dazzling objects.

4th. *Proximate Cause.*

"The proximate cause of weakness of sight, instead of being a local debility, consists in an exquisite irritability and sensibility of the

---

* I am assured by an intelligent and learned traveller, that the inhabitants who reside at the foot of the Glaciers in Switzerland, acquire this habit in a remarkable degree, in consequence probably of the vivid light that is reflected from the accumulated fields of ice and snow, to which they are perpetually exposed.

† Many people amongst the Æthiopians, Africans, &c. who have an extreme degree of tenderness of sight, owing to the great brilliancy of the Sun's rays, suffer exceedingly, according to Haller, (Tom. v. p. 490.) from violent epiphora, or watery eye, which in fact renders them nearly blind during the day.

retina, retina, the effect of great turgescency of the vessels, or a chronic inflammation of that membrane, or the choroid."

5th, *The Cure.*

The remote causes of this disease, or what have been understood to be its remote causes; as well as an obvious reasoning upon its exciting causes, led to an erroneous conclusion, and to a fundamental mistake in the treatment.

A morbid sensibility of the retina, the effect of exhausted nervous energy, was deemed to constitute the very essence of this complaint. On this hypothesis, the symptoms were combated by the external application of sedative and tonic, and by the internal exhibition of corroborant and nervous remedies.

The compleat failure of this method led Mr. Stevenson to adopt an antiphlogistic and evacuating plan. His observations brought him to conclude,

"That the exquisite sensibility of the Eye might with more probability result from a chronic inflammation, or at least a highly turged condition of the blood-vessels of the retina or choroid."

"Agreeably to this supposition, the indications of cure must consist, not in giving additional tone by the use of cold astringent applications, and internal strengthening medicines, but in lessening the plethoric state of the vessels of the posterior membranes of the eye, and in obviating, at the same time, the exquisite sensibility of the retina."

On this principle, leeches were applied* to, generally, the

---

* "The application of leeches so immediately in the vicinity of the eye, has been pointedly reprobated by a celebrated author on ocular complaints, because, says he, they have sometimes been found to occasion a considerable swelling of the lids, and have also, for a time, increased, instead of lessened the irritation of the eye. In order to prevent which mischief, he adds, it will be proper to apply the leeches to the hollow of the temple. In a very large number of cases in which I have known them applied to the lower eye-lid, under the direction of the late Mr. Saunders, as well as under my own, I have indeed very rarely witnessed the effects above alluded to; and where any inconvenience of the kind has occurred, it has been in highly irritable habits greatly disposed to erisypelas, in which cases the same consequences are apt to supervene even from the application of a blister. But I am convinced, from the fullest observation and experience, that *the benefit they afford the patient when placed as near as possible to the inflamed eye, infinitely exceeds what the same number are capable of yielding, by being laid on the temples,* and more than compensates for the greater degree of ecchymosis, which I admit is more prone to take place by the extravasation of blood into their loose reticular texture, than into the denser cellular membrane of the temples, and which temporary inconvenience constitutes the under eye-lid; cathartics were employed, and managed in a way which Mr. Stevenson explicitly describes:† Fomenta-

---

the principal, if not the only real objection against their application to the lower eye-lid."

Having ourselves observed the inconvenience here stated, and also a troublesome ulceration when leeches have fixed on the upper eye-lid, we are gratified in stating the opinion of a gentleman of much practical knowledge, on a point often important in private practice, in more than one view. When an ecchymosis does take place, Mr. Stevenson recommends aq. ammon. reduced with water, or the juice of *Solomon's seal*, for removing it. We have often had an opportunity of observing the advantages that arise from attending to *euporistic* medicines. The Solomon's seal (*Convallaria multiflora* Linn.) affords an instance of an indigenous plant that deserves investigation. It is so common as to be found in the woods of many, if not all the English counties; is perennial, flowers in May and June; and has been long known, as a popular remedy for removing ecchymosis very speedily. The professors and pupils of the pugilistic schools are well acquainted with this fact. It may be worthy of enquiry whether the reputed effect of *Convallaria multiflora* in hernia, has any actual foundation. Applied to the protruded part, and given internally in powder and infusion, it was once thought to be useful. If mixed with its congenors the *Convallaria maialis*, *C. verticillata*, and *C. polygonatum*, would its properties be improved?

† "Purgative medicines as exhibited by practitioners in general, for the acute inflammation of the eye, whether external or internal, have been given, much *too sparingly*, both in regard to their dose, and to the times of their repetition. If they be meant to produce any decidedly good effect, they must at first be administered in a full dose, so as to excite an immediately copious evacuation, and repeated afterward in such a way as to keep up a conssant determination to the bowels. Should the patient be of a robust habit of body, nothing is so effectual for that purpose as a full dose of calomel with antimonial powder, and in two hours afterward, a sufficient proportion of powdered jalap, and double the quantity of cream of tartar in mint water, or gruel; by the operation of which, a large discharge of serous fluids is solicited, and the vigor and activity of the general circulation sensibly diminished. In more delicate constitutions the calomel must be given sparingly, and in lieu of the jalap, an adequate quantity of *magnesia vitriolata* in infusion of senna."

"I have repeatedly observed, Mr. Stevenson adds, that gentle aperients in these cases make no impression upon the disease; but, if the operation of the remedy be powerful, the most manifest advantage is certainly obtained. In the management of this class of remedies, it is customary to allow two or three days to intervene before they are again repeated, by which time the effects of the first dose have wholly subsided, and little comparative advantage is gained. On this account the cathartic should be employed in a full dose, and as frequently as the patient can bear.

used warm, and composed of narcotic substances, in an aqueous decoction*. These were applied morning and evenbear. The object is not only to lessen the actual quantity of circulating fluids, but also to prevent their immediate accumulation, and the consequent distention of the vessels upon which the inflammation depends. This purpose should be effected by the observance, at the same time, of a strictly antiphlogistic regimen, allowing only a small quantity of diluting liquids, and by interposing between the active purgation, small quantities of emetic tartar, combined with some mild saline aperient, repeated so as to keep up a constantly moderate diarrhœa." In every species of acute ophthalmia, and in every kind of inflammation, or fullness of the vessels of the eye, Mr. Stevenson found this method effectual; and often superseding the necessity of repeated topical bleedings. This practical fact, so fully confirmed by Mr. Stevenson's experience, was undoubtedly known to HIPPOCRATES, who asserts, Aphorism 17, section 6. *Lippitudine laborantem alvi profluvio corripi, bonum;* and succeeding practitioners have availed themselves of it. Celsus says, *omnique dejectio lippiendi prodest.* (Lib. 4, cap. 8, page 77, 8vo Edit. Biponte, 1786,) and the annotators on Hippocrates subjoin, "*Ratio est, quia fit revulsio a supernis deorsum materiæ sive humoris ophthalmiam patientis.* Debet ergo Medicus naturam imitari, cum illa morbum sanat. *Cardan. p.* 622, *Conf. Duret. in Coac. p.* 120. *Enim vero optime caput ab humorum affluxu liberant laxantia, veletiam pro circumstantiarum ratione purgantia. Et Galenus gravis testis est, nonnullos, quibus oculi inflammati, sola purgatione per alvum una die sanatos.* Hoffm. Med. Rat. T. 4, S. 1, p. 525. We believe, however, that the fact has never been so strongly put as by Mr. Stevenson.

* For the fotus Mr. Stevenson employed the capsule of the poppy, chamomile flowers, rosemary, and eyebright *infused* in hot water, but confesses he is unable to determine if any of these substances augmented the efficacy of the water. Are we to suppose the *Euphrasia officinalis* was employed because it was formerly in repute as a remedy for impaired vision, and even had the power to make old eyes again young. *Tantum esse, ait, Euphrasiæ in imbellicatæ visus vim atque efficaciam, ut ejus usu septuagenari os quosdam, propter vigilias et studia plurima amisso visu, in decrepita ista etate, cum recuparasse observaverit.* These are the tales of other times. We have more satisfaction in resting on the strong evidence of the efficacy of the long neglected digitalis. I cannot omit to state, says Mr. Stevenson, (p. 77) that I have used a warm decoction of digitalis *(pupurea)*, with the effect of diminishing, materially, the exquisite sensibility of the eye; nor does its application produce any uneasiness, like the different preparations of opium. In violent inflammations of the eye, the decoction of this plant has been used with marked success by Dr. Haworth;—in the most severe attacks of the Egyptian ophthalmia it has alleviated the suffering—and it has been extensively employed, in strong infusion, to the inflamed eyes of horses, with similar results. Saffron in the form of a warm infusion tends also. Mr. Stevenson assures us, most powerfully to take off the morbid sensibility of the eye.

ing, as hot as could be comfortably borne; and after the eye was dried from the fomentation, a quantity of a *tinctura opii mitis** was dropped into it.

But sometimes, in very confirmed cases, it became requisite to apply a permanent blister to the head, below the ears, or between the shoulders.

Under this management modified to the peculiarities of individual cases, the disease usually subsided in a very short period; and due perseverance, with the addition of tonics, when the congestion was removed, effected permanent cures.

There are diseases of the eye so nearly resembling this as to be mistaken by very able men. It is essential to clearly ascertain these, because the remedies proper for them will be useless in "*weakness of sight*," and *vice versa*.

"By the disease termed by Hippocrates, amblyosmos; by Aretæus, amblytes; by Ætius, visus debilis; by Boerhaave, visus hebetudo; and by some French writers, vuë confuse, faiblesse de la vuë, and mauvaise vuë is not to be understood the same complaint which is described in this Dissertation, under the name of *weakness of sight*, but rather an indistinctness of vision, or absolute and complete ambliopy.†

This ocular complaint, properly called *Dulness* of sight, I must briefly

---

* The following formula is given for this tincture. R. *opii purificati, croci anglica.* āā ℨij. *spir. gallic. alb.* ℥j *aquæ distillatæ* ℥vij. *macera in vase clauso per sex dies, deinde tincturam cola.*

The patient being placed in a supine posture, with the head somewhat elevated, and the eyelids gently closed, a little of this tincture should be poured into the inner canthus of the eye, from whence it should be suffered gradually to insinuate itself between the palpebræ, by inclining the head to that side, and, at the same time twinkling the eye-lids; avoiding, in the interim, most carefully, every rude effort forcibly to separate them. It may be applied by means of a camel hair pencil, in the following manner. The operator, with the fingers of one hand, must cautiously depress the lower eye-lid, by which it will be somewhat averted (inverted), when the pencil, fully charged wlth the tincture and held in the other hand, is to be rapidly and dextrously swept across its inside, permitting the eye-lid instantly to resume its proper situation: p. 82. It seems an object to accurately diffuse the tincture over the whole anterior surface of the globe of the eye, and in particular to bring it in contact with the whole of the cornea. A rude application of this tincture, by forcibly pulling open the eye-lids, and dropping it upon the centre of the cornea, increases the pain, and adds to the irritation.

† Ambliopia est visûs debilitas sine admodum visibili oculi vitio. Myopes et presbytes in certâ objecti distanciâ solummodô confusè vident: nyctalopes et hemeralopes certo diei tempore tantùm malè vident, át ambliopes in quâvis distanciâ et quôvis diei tempore objecta debilitèr discernunt. Plenck Doctrina de Morbis Oculorum, p. 186.

notice, because I have known it confounded with *weakness* of sight, although it is in fact the very reverse; and depends not upon an excess, but a positive want, of sensibility of the retina.

The principal symptoms are, not only frequent alterations in regard to the precise limits, but also a great indistinctness and confusion of vision; under all circumstances of time and place. If the eyes be much exerted, they soon become fatigued, which renders it necessary, every now and then, to close and gently rub them, when the patient can again see somewhat better for a short time. The eyes appear dull and inanimate. The iris, which is more or less dilated, is susceptible of very feeble, oftentimes scarcely any motion, even on the sudden impulse of a strong light, which occasions *very little* uneasiness. This malady is most apt to attack persons who are past their meridian; and is generally brought on by the too free use, or rather abuse, of the organ of vision, co-operating with other causes which have a tendency to debilitate the general and nervous system. The disease not unfrequently remains almost stationary for a great length of time. In other instances its progress is much more rapid, when it generally terminates in complete gutta serena, or total blindness.

Various and extremely contradictory remedies have been recommended by oculists for the cure of this very formidable complaint; which, however, I shall forbear at this time enumerating. The first thing to be attended to is, to allow the eye as much rest as possible, and to avoid particularly what may be considered the exciting causes of the disease. It is, I believe, in many cases absolutely incurable: and the only remedies which I have ever known to prove beneficial, are topical stimulants, as * electricity, galvanism, æther, infusion of capsicum, rubefacients applied to the palpebræ, &c. whereas, in *genuine weakness* of sight, these means are certainly useless, some of them highly hazardous. Internally, too, mercurials given so as slightly to affect the mouth, together with the arnica montana, and deobstruent medicines, have sometimes been productive of benefit. Double convex glasses, by concentrating the rays of light, never fail to afford, in this case, considerable assistance to the sight.

It is this particular species of disease to which I presume Mr. Ware alludes in the following passage. "I cannot omit to mention," says he, (Vol. I. p. 122) "that in some instances where the eye has been particularly weak," (a term he employs in a vague and indefinite manner), "without any perceptible cause to produce it, the application of spirituous remedies that have been highly rectified, such as the medicine sold at Riga, under the name of the Riga balsam, or the æther of the Lon-

* I think it consonant with the present discussion to remark, that some cases which were deemed instances of gutta serena, I have ascertained to be examples of actual dulness of sight, and that they derived the most essential assistance from the application of the electric and galvanic influence. And I cannot forbear to add, that a few cases of blindness, which have fallen under my observation, and which were likewise ascribed to a paralytic state of the optic nerve, were altogether sympathetic affections, depending upon visceral irritation; by the removal of which the patient has obtained a complete cure. Of the latter description are the successful instances of amaurosis, related by Richter, Schmucker, and Scarpa.

don pharmacopœia, either alone or mixed with an *equal proportion of sugar and water*, has sometimes been greatly useful. In a few instances also, the excitement of a violent inflammation, by the application of other stimuli, has been found of use to overcome the enfeebled action of different parts of the eye."

Dulness of sight again differs from the Glaucoma, as there is not that deep-seated grey appearance, or shining pearl colour, observable in the latter complaint.

I am well aware, and have met with many examples of a slight species of Psorophthalmy, which, if only cursorily regarded, seems, in some respects nearly to resemble weakness of sight. Mr. Ware, indeed, appears, from the cases he has published, to have actually identified it with this complaint. I think it necessary, therefore, to dwell a little upon this topic; and I doubt not to be able to prove, by contrasting their respective symptoms, and the different modes of cure they individually require, that they are in reality perfectly distinct ailments. The description of this affection of the ciliary glands is so accurately given by that respectable author, that I shall take the liberty of transcribing the particulars, and of subjoining a few remarks in support of my opinion.

"The psorophthalmy," says Mr. Ware, (Chirurgical Observations relative to the Eye, Vol I. p. 116), "not unfrequently occurs, without producing the slightest appearance of inflammation, either in the eye or eye-lid. I have attended a very considerable number of such cases; and in many, the only intimation of the nature of the complaint has been derived from the description given by the patients themselves. Whenever I am informed that the edges of the eye-lids have a disposition, be it ever so slight, to adhere to each other after they have been long in contact, as during the time of sleep, and when this is accompanied with an uncomfortable sense of weight in the lids on the approach of night, in consequence whereof the patient involuntarily shuts them without being drowsy, and without any particular stimulus being applied to the eye to give it pain, I always suspect that the secretion of the ciliary glands is in a diseased state; and in many such cases, I have found the success attending the use of the unguentum hydrargyri nitrati, recommended for the cure of this disorder, quite as effectual as in those other instances, where the excoriation and redness of the eye-lids have been visible on the slightest inspection."

There are, however, several symptoms above mentioned, which decidedly characterize that species of diseased ciliary glands, and which serve at all times to distinguish it from the subject of this Dissertation. I allude to the uncomfortable sense of weight in the lids on the approach of night, the tendency of the tarsi to adhere together during sleep, and the involuntary disposition to shut them without being drowsy, and without any particular stimulus being applied to give them pain.

In the complaint in question, on the contrary, the meibomian glands perform their functions in the most perfect manner, consequently there is not any sense of weight of the palpebræ towards the close of day, inducing a propensity to shut them independently of drowsiness or uneasiness, nor is there any adhesion of their edges during the night. Hence, the application of mercurials to the tarsi, which are almost specific in the psorophthalmy psorophthalmy are absolutely useless, often prejudicial in genuine weakness of sight. Besides which, the acute pain excited by the admission of a vivid light to the eyes in cases of the latter disease, furnishes a striking discriminating feature, sufficient to point out a decided difference between weakness of sight, and the above described slight species of psorophthalmy.

The injuries done to the delicate structure of the organ of vision by the improper treatment of many of its complaints, especially those of the minor class, by their falling to the care of empirics, and nostrum-mongers, or even under gentlemen regularly instructed, and sufficiently qualified in the general duties of the profession, are very numerous. These often arising from topical applications, we cannot doubt the propriety of giving a general currency to Mr. Stevenson's observations on the subject.

" To a person conversant with ocular complaints, it is really a matter of astonishment to witness the gross mistakes that are perpetually made in the selection, and proper management of external applications for the eye. For, nothing is more common, than to see astringent lotions resorted to in the very *early* stages of Opthalmia, by which the inflammation and pain become speedily and excessively exasperated.— Whereas, if they had been withheld till the secondary symptoms had commenced, their utility would then have been as strikingly displayed, as in the former instance was the mischief they occasioned. Indeed it has been doubtless owing to the *casual* employment of the tonic class of medicines, during the *chronic* state of inflammation, that many boasted nostrums of this description are indebted for their reputation, in the cure of morbid affections of the eye. I should not, probably, be incorrect in asserting that, more injury has resulted from injudicious officiousnesss in this particular, than from all other measures which have been adopted for complaints of that organ. Innocent as a common poultice is generally esteemed, I have, in several instances of Ophthalmia, seen it applied with the lamentable effect of causing, in some instances, the formation of abscesses between the lamellæ of the cornea, which not unfrequently end in incurable blindness, by the opacity they leave behind them; and in other cases, it has occasioned a speedy ulceration, and consequent rupture of the cornea, and staphyloma. In fact, a good remedy *untimely* applied to the eye, has often rendered its diseases, which were at the time scarcely uneasy, absolutely incurable. And I am persuaded that, less mischief would in general arise, were every kind of application for the eye withheld, than from employing improper ones. And thus it has scarcely ever happened, that an appropriate collyrium has been used for the disease under consideration. Amongst the different formulæ prescribed for it, the Zincum vitriolatum has almost invariably been the efficient ingredient, on the supposition doubtless, that astringents alone were required with a view to brace the fancied relaxation of the organ. From the amplest experience of the effects of that plan I dare maintain, that till the plenitude of the vessels is diminished by proper evacuations, it uniformly does harm. Indeed, I have really my doubts, whether eye waters are at all desirable, during the existence of the great tenderness

L 2 of of sight. If any are advisable, I would recommend such only as are calculated to allay action, and appease pain, as the different preparations of lead; of which probably the best is composed of the cerussa acetata gr. j. dissolved in ℥j. elder flower water, and rendered perfectly limpid by the addition of a few drops of distilled vinegar. When used, it should be made a little warm, by immersing a cup, containing some of it, in a bason of boiling water. For I must repeat, that *cold* applications manifestly do harm in the *early* stage of this complaint, probably by constringing the exhalent pores of the cornea, and by propelling the blood from the superficial, to the deep-seated vessels of the eye, already in a preternaturally turgid condition. And, if any good effects are to be derived from the lotion, it must be repeated frequently during the day.

I trust I shall be pardoned for the digression, if I cursorily make a few practical remarks on the composition of eye waters. According to the manner in which they are occasionally prescribed, I am convinced that they are mischievous, when employed in cases of ophthalmia, or of great irritability of the eye. I allude to the custom of making them with the oxyds of metals, or other rough and insoluble powders, or with liquids, which though pellucid individually, yet when mixed together, suffer an immediate decomposition, and in consequence, the eye water is rendered more or less turbid and inert. Indeed, nothing is more common than to see the cerussa acetata dissolved in common water, by which the lotion becomes instantly opaque, and the greater part of the lead is soon precipitated in form of a whitish calx. Must not the rough ingredients above alluded to, stimulate the exquisitely irritable cornea in some degree like so many heterogeneous particles of sand? Sometimes the tinctura, or the vinum opii, is added to an aqueous vehicle, the resinous particles of which separating from the watery menstruum, they are introduced into the eye, and adhering to some part of the globe, excite in it no small degree of irritation. However refined this may appear to the inaccurate observer, I must declare that, I have personally witnessed very acute pain having been brought on by this apparently trivial cause, which has not ceased, until the removal of these little filaments of opium.*

In a word, I hold it to be an invariable maxim, that eye waters should be rendered *perfectly transparent* before using, either by the addition of an appropriate solvent, (if the active ingredient be derived from the mineral kingdom, taking care that it coincides with the intention of the remedy), or else by filtration through paper.

But although I am not strongly prepossessed in favour of any kind of collyria, from a satisfactory experience of their utility in weakness of sight, *before* the exquisite sensibility of the retina is relieved by the plan of depletion above recommended, yet *after* this object is once fully attained, the vessels of the eye will *then* require astringents to impart increased tone and energy. Otherwise, a relapse would be very apt to occur, from the blood again accumulating in vessels, relaxed by long continued over-distension.

Of the applications best adapted to accomplish that purpose, I may name a weakish solution of Zincum vitriolatum, dissolved iu camphorated mixture, or in rose water, and filtered. But I think a still more

* I have met with similar effects from neglecting to pass an infusion of digitalis, prepared with fine powder of the plant, through linen cloth before using it.

Of effectual collyrium is formed of one drop of the sulphuric acid, and an ounce of distilled water, to which may be added a few grains of Zincum vitriolatum, and a small quantity of brandy. Nor, is a solution of the argentum nitratum, in the proportion of from half to a whole grain to one ounce of distilled water, and half, or a whole drop of the nitrous acid, a contemptible tonic collyrium in this case. Even cold spring water is a remedy, by no means destitute of efficacy in this stage of the complaint.

The above eye waters, being intended to act as corroborants, should of course, be applied *cold*, three or four times a day.

As Mr. Stevenson's treatise is more of a practical than a speculative nature, and contains some novel methods of managing a disease which seems to have been much misunderstood, we do not hesitate to recommend it to our readers.

When our Analysis was going to press, we received the following Letter :—

GENTLEMEN,

I take the liberty of requesting a place in your very respectable Journal, for the following observations upon a publication of Mr. Stevenson's, upon the disease called weak sight. My sole wish, in communicating these remarks, is to do justice to a most respectable member of the profession, whom I have long known, and also to set some other matters upon their proper footing. In attempting to effect these objects I should be sorry to shew any disrespect towards Mr. Stevenson.— The first observation which I shall make upon the dissertation, is, that from the language used in page 5 of the preface, the reader might conclude that the knowledge of *the proximate cause* of the disease in question, as well as *the plan of cure*, originated with Mr. S.—"I was," says he, "repeatedly disappointed in any attempts to relieve it, before a different view of the proximate cause, and a corresponding variation in the mode of cure *suggested themselves*." This view of the proximate cause is described by Mr. S. in the following words : "And with regard to the proximate cause of weakness of sight, *instead of local debility*, *I will hazard the opinion*, that it consists in an exquisite irritability and sensibility of the retina, the effect of a great turgescency of the vessels, or a chronic inflammation of that membrane, or *the choroid*." Mr. S. however, in the next sentence, quotes this opinion from Sauvages : "Intolerantia lucis retinæ sensibilitatem adauctam esse probat, sive detur ejus infarctus phlogisticus, sive tensa sit nimium choroidea, ejusque expansio uvea." This quotation from Sauvages, appears to me to convey nearly the same opinion as that suggested by Mr. S. But leaving the reader to judge for himself, I shall proceed to say a few words upon the corresponding variation in the mode of treatment, which suggested itself to Mr. S. at the same time. In this point, also, or at least, in the chief part of it, Mr. S. it would appear, had been long anticipated by Mr. Ware. To make this clear I need only refer the reader to the cases quoted at length from Mr. Ware's observations. From these cases (which extend from page 51 to 58, inclusive, of Mr. S.'s work) it is evident, that *evacuation by means of leeches* was used by Mr. Ware, when the plan which he usually adopted, proved ineffectual.—Independent, however, of these considerations, the general plan recommended by Mr. S.

is is by no means new. I am acquainted with many eminent practitioners in different quarters of the kingdom, who occasionally resort to leeches, but uniformly employ the other parts of the evacuant plan, such as purging with calomel, blisters, setons, &c. They have also used remedies to promote a determination to and discharge of blood from the nose, of which Mr. S. makes no mention. To those surgeons, however, who were ignorant of the evacuant plan, Mr. S.'s statement of the fact will be acceptable. Valeat quantum valere potest.—Having now done justice to the celebrated Sauvages and Mr. Ware, I shall next say a few words respecting the validity of the proximate cause. Mr. S. has, however, stated it in terms so different in different pages of his work, that it will be necessary to collect the whole into one point of view. After describing this cause in the words already mentioned, (page 11) Mr. S. next remarks, (page 12) "that this highly nervous and vascular tunic (the retina) is *either* in an actually inflamed, *or, at least*, in a morbidly *distended*, and, consequently, irritable condition." He next observes, (page 12) "that *genuine weakness of sight* is actually a disease in the retina arising from the great turgescency, or chronic inflammation of that membrane," &c. Next (page 33) "the exquisite sensibility of the eye might with more probability be the result of a chronic inflammation, or, *at least*, highly turgid condition of the blood-vessels of the *retina* or *choroid*." Next (at page 60) "The proximate cause of weakness of sight is a turgescency or *more* or *less* inflammatory affection of the *posterior vascular membranes* of the eye." Soon afterwards (page 62) is this expression, "Do not the history of the above case and its dissection *demonstrably* prove, that *weakness of sight* does not, *always at least*, proceed from a nervous affection of the retina, but that it is the *actual* result of a greater or less degree of inflammation of the choroid ?" Lastly, our author observes, (page 29) "In these cases (of psorophthalmy) the inflammation seems continuous, extending itself from the margin of the tarsi along the PALPEBRAIC to the CORNEAL conjunctiva. Even in this instance, however, I conceive that the intolerance of light *does not arise, at least* only sympathetically, from an affection of the retina, but rather from the cornea itself, which" &c. &c.—From the quotations it appears, that Mr. S.'s opinion respecting the proximate cause is somewhat fluctuating. For my own part, I think it questionable and inconsistent with facts, but to attack it under all its protean forms would be a fruitless task, or almost impossible; for I confess myself ignorant of Mr. S.'s meaning when he talks of the *distended condition* of the *turgescency*, or of the *nervous affection of the retina*, or *of the posterior vascular membranes of the eye*. Still less can I comprehend how "light really acts in expanding the vessels of the retina;" (page 16.) That blood may expand, or distend these vessels, is obvious: but we have yet to learn what expansive powers are exerted upon blood-vessels by light, even from the meridian sun, which as our author informs us (page 15) destroyed the Roman general Regulus. I shall therefore confine myself to Mr. S.'s first opinion.

The first observation, which I shall make, is, that Mr. S. in defining his proximate cause, supposes that exquisite irritability and sensibility of the retina, is the effect either of chronic inflammation of the *retina*, or of the *choroid* indifferently. Why chronic inflamation of the *choroid* should produce produce the same symptoms, as a similar affection of the *retina*, since the two membranes are totally different in their structure and uses, requires elucidation. My next observation is, that the chronic inflammation of any membrane or organ, with which I am acquainted, is with difficulty and only gradually removed. This does not well accord with the *astonishing* and *immediate efficacy* of leeches and evacuations. Nor does chronic inflammation ever exist long without leaving lasting marks behind it. How few anatomists, however, have detected any obvious morbid change in the *retina* or *choroid*.

But admitting that chronic inflammation, or a great turgescency of the vessels of the retina or choroid coat exists, it is somewhat remarkable that these affections do never spread to the iris. The blood-vessels of the iris are most intimately connected with those of the choroid, yet the latter may labour under great turgescency, without producing any perceptible change in the former. The same observation may be made with respect to one part of the vessels of the sclerotic coat. These are derived from the same source as the vessels of the choroid coat, nay some of the vessels of the choroid itself pass directly through the sclerotic coat, and ramify upon it externally, yet, in the disease in question, no symptoms of turgescency appear in these vessels, however close their connection. For these reasons I consider Mr. S's opinion highly questionable: and if I may be allowed to advance another, I would say, that weakness of sight is, probably, produced in this manner. In the first place, the exciting causes, such as excessive light, intense reading, &c. being frequently applied, the sensibility of the retina is increased, which induces some degree of increased action in its blood-vessels, and those of the choroid also, by sympathy. This state would, in most cases gradually subside, were the exciting causes withdrawn. From the repeated application, however, of these causes, a morbid action at length arises in the *secretory vessels* of the choroid. This so far alters the quality or quantity of the secretion of pigmentum nigrum, as to render it unfit for its purpose of absorbing erratic or reflected light within the eye. In short, I conceive, that the state of the eyeball in this disease, bears a striking analogy to that defective state, which obtains in albinos.——Such a state, I apprehend, perfectly coincides with the effect of the evacuant mode of treatment, and is liable to no material objection.——The effect of the first application of leeches is probably that of allaying the irritability of the retina: this topical evacuation, with the use of sedative applications, and the removal of all exciting causes is principally instrumental in suspending this most distressing symptom of the disease, and at the same time lays the foundation of that healthy change in the action of the secretory vessels of the choroid, which is finally completed by the use of purgatives. The morbid action in the secretory vessels of the choroid, in this instance, is, probably, not inflammatory, but rather bears an analogy to that change, which occasionally occurs in the action of the vessels of other secretory organs, producing an alteration in the nature of their secretions, without being accompanied by any symptom of turgescency or inflammatory disposition. The effects of purging and other evacuations, in re-establishing their healthy functions, is well known. This view of the proximate cause explains also, why tonics, conjoined with sedative applications, and freedom from all exciting exciting causes, are, in particular habits, effectual in removing this disease; particularly if conjoined with alterative doses of calomel.

I shall not dwell longer upon this subject, as I am, by no means, very solicitous about the adoption of this or that opinion respecting the proximate cause of weak sight. What is of much greater importance, is, that Mr. S. setting aside all selfish motives of concealment, has candidly made known to the profession at large his experience in means, which few practitioners have employed more extensively or successfully, than himself; and that he has, moreover, announced some important improvements in the treatment of *Blear-eye*, which, after repeated trials, he at length discovered (See note, page 28). That the public may lose no part of the advantage to result from this discovery, I dare say, that the Editors of the Medical and Physical Journal will be happy to place it without loss of time amongst their excellent pages.

I am confident that Mr. S. will pardon any liberty I may have taken, in making these remarks upon his publication. To do justice to all parties has been my sole aim, and I know that no member of the profession would with more pleasure "reuder unto Cæsar the things which are Cæsar's."

I remain, Gentlemen,

Your obliged servant,

CHARLES HUMPHRIES.

*Shrewsbury,*

*December* 6, 1810.

---

*A Treatise on the Venereal Disease. By* JOHN HUNTER. *With an Introduction and Commentary; by* JOSEPH ADAMS, M. D. Author of Observations on Morbid Poisons, &c. 8vo. London, 1810. pp. xvi.—600. six plates.

WE should not have thought it necessary to notice a third edition of this valuable work, had it not been for the illustrations of one who has so long been considered the interpreter of his favourite master. To pursue the plan we have hitherto adopted, we shall content ourselves with offering a few extracts as a specimen of the work, instead of the often unthankful task of minutely criticising individual passages.

The following is Dr. Adams's commentary on that part of Mr. Hunter's introduction which relates to hectic fever.

"The hectic fever, till Mr. Hunter's time, was usually considered as the effect of matter absorbed. Phthisis pulmonalis and psoas abscess seemed to furnish proofs of such a cause. The late Dr. Heberden has, however, a very ingenious paper on hectic fever, making the first article of the second volume of Medical Transactions. This accurate writer seems rather to consider the disease as the effect of repeated formations of matter, than of its absorption. He calls it the symptomatic, the irregular intermittent, and the fever of suppurations. The disease itself is most admirably described by him, but some parts of the pape paper are confused, from the difficulty the author found in tracing the cause constantly to suppuration. Hence he describes the various symptomatic fevers as making different forms of the hectic. Thus the shivering from the first formation of matter, and the high feverish irritation sometimes consequent on a wounded tendon, are all inncluded in the hectic, which would be reasonable enough, if the *symptomatic* fever always became *hectic*, which we shall presently see is not the case. Mr. Hunter, though he considers both as the effect of sympathy, yet distinguishes them as arising from different causes, and exhibiting different phenomena. The *symtomatic* fever is usually acute, and arises from, or is only a *symptom* of some acute local injury, which, throughout all its stages, whether in its commencement or its progress to suppuration, is attended with shivering and cocsequent fever. Sydenham has well marked these stages in small-pox. They occur in every common abscess, if the progress is rapid.

*Hectic*, or (if we translate the word) *habitual** fever, is less acute than the symptomatic, but more permanent in its returns. As it almost always attends phthisis pulmonalis, and large incurable abscesses, it was supposed to arise from the absorption of matter. But this error might easily have been removed by reflecting, that in large abscesses matter is sometimes absorbed without injury to the constitution. Suppuration is one of the curative processes of nature, in parts which cannot return to their original actions, and like other new actions is usually ushered in by shivering, and consequent fever. But where suppuration is unattended with inflammation, the constitution is little affected, so that no shivering or consequent fever arises. Thus in the psoas abscess, whilst it is continually enlarging by an increase of matter, or by fresh suppurations, the constitution is very little affected, and in the lungs large tubercles are formed, which suppurate, and whilst the matter remains in the capsules, the constitution only suffers from the loss of so much lung. But as soon as the abscess is opened by nature or art, or the matter of tubercles finds its way to the bronchia, inflammation takes place over the whole surfaces, as the first means by which the part is to be restored; and this inflammation is attended with shivering and heat, constituting a paroxysm of fever. If this attempt made by nature to restore the part is ineffectual, she renews it; but this and every future attempt are often insufficient to relieve so much mischief as the parts have sustained. Still, however, the attempt continues as long as the constitution retains power to excite inflammation, and each attempt is attended with a similar paroxysm. Hence the fever becomes *habitual* or *hectic*, not from the absorption of matter, but from the repeated attempts at restoring parts which cannot be restored. This will be further illustrated when we arrive at Mr. Hunter's doctrines concerning *lues venerea*.

Thus the symptomatic and hectic fever are both of them similar, in-

---

* The Greek word ἑκτικος can scarcely be translated by any other word than *habitual*. In both languages it is derived from the same root, from εχω *habeo*, ἑξις *habitus*, and ἑκτικος *habitu positus*, or as we say in English, habitual. Habitual, though there is no such classical word as *habitualis*, must be derived from the Latin habitus, from which we vernacularize habit.

asmuch as they arise from the *sympathy* of the whole constitution with a local disease, and the only difference is, that the former is for the most part more acute, and rarely returns more than once or twice, after which, if matter is formed, it is either absorbed, or if it comes forward to the surface, granulation succeeds, to restore the lost part. The hectic, on the contrary, is less violent, in proportion as the constitution is previously debilitated, but returns as long as strength enough remains to attempt the curative process. To bring these more immediately to the present purpose—symptomatic fever, in swelled testicle, is the sympathy of the constitution with the violent inflammation excited. Hectic fever, in *lues venerea*, is the perpetual attempt of the constitution to cure a disease, or to excite such a new action as will supersede the disease: but we find, by experience, that the constitution is unequal to such an attempt. The attempt, therefore, not having succeeded, is renewed till it becomes *habitual*; or, in other words, "*hectic* fever is an habitual universal sympathy of the constitution, struggling with a disease which it is unable to overcome."

The following passage we insert, not only on account of the credit it does our commentator, but inasmuch as it contradicts an accusation too often retorted from one nation to another, without sufficient enquiries by either. Among the arguments to prove that "the poison is the same in gonorrhœa and chancre," Mr. Hunter has introduced one taken from the supposed introduction of the disease into the South Sea Islands. At the close of the commentaries on this section we have the following remarks.

"Having thus, says Dr. Adams, I hope sufficiently illustrated Mr. Hunter's explanation, how the same matter applied to different surfaces may produce different effects, the reader must indulge me with a few words on the South Sea disease.

"About the year 1800, a lady of fashion who was recommended to my care in Madeira, brought with her the French account of De la Peyrouse's voyage. Though I had leisure enough to peruse the whole, yet the letters of his surgeon attracted my particular notice. After examining them with the greatest attention, I could not help remarking that he wrote of *mal venerienne* without the precision of a Hunter. In the end, I was convinced there was reason to doubt whether De la Peyrouse's surgeon had met with the venereal disease in any of the places in which he spoke of it with so much freedom. This induced me to examine the accounts of Captain Cook's voyages; and the result was, a thorough conviction, that, if the venereal disease existed at all in the South Sea islands, there was at least no satisfactory proof of it. Under this impression, I wrote to three physicians in London, explaining my doubts, and, perhaps with more Quixotism than prudence, was willing, if encouraged, to make a voyage in order to ascertain a point involving not only an important medical question, but in some measure the national reputation.

Fortunately, this question has been much better decided, by one who candidly admits his arrival at those islands with a most perfect conviction that the disease existed there in all its forms. His inquiry was not, not, therefore, whether he should find it, but how general, and with what severity, it would appear, and also how he might preserve the health of his crew. From these circumstances, and still more from the character of the gentleman, no doubt can be entertained of the faithfulness of his conclusion, which is, that "*the venereal disease is unknown in Otaheite*."* At first sight, it may seem strange that this opinion of mine has never been published before the fact was confirmed by Mr. Wilson. To this I can only answer, that to offer an opinion on a subject without the means of ascertaining it, must, at least, be premature. There are, however, fortunately, witnesses that such was my opinion. Dr. Garthshore is one of the gentlemen to whom I wrote from Madeira on the subject. The late Dr. Pitcairn was another, which is confirmed by his note now in my possession, and also by a communication he made to a most distinguished philosophical character now living.

But perhaps it may be asked, "Admitting the whole as I have stated it, why should the reader be troubled with the account?" In order, I answer, that he may learn there are certain characters by which the venereal disease may be distinguished with certainty; that these are so well marked as to be understood by description; and that even the absence of them may be ascertained by those who take the trouble of examining with sufficient diligence. It may then be asked, how could the accurate Hunter have fallen so easily into the belief, that the venereal disease was known in all its forms in the South-Sea islands. Mr. Hunter, it may be answered, had not the prolixity of a French surgeon's account to make him doubtful on the subject. When I speak of prolixity in this case, it is not from disrespest. Though De la Peyrouse's surgeon was not mistaken, still his descriptions are so minute as to enable the reader to comprehend what symptoms were present. It was from the description, not from the name, of the disease, that I suspected De la Peyrouse's cases were not venereal, and it was natural to transfer this scepticism to the South Sea disease. On examination, it was found, that the account, defective as it is, would authorize the same conclusion.

These extracts are enough to shew the nature of the performance, which is all we profess to do in a work, of which so large a portion is occupied by original communications.

---

* "From the foregoing sttaement," says Mr. Wilson, "it may be concluded, without, I hope, presuming too much, that notwithstanding the melancholy accounts we read of the ravages of lues venerea at Otaheite, and even disputations about its first importers, this disease was not introduced there antecedent to the Porpoise's voyages."—See *Edin Med. Journal*, vol. II. p. 283. The Porpoise is His Majesty's ship, of which Mr. Wilson was surgeon, and arrived at Port Jackson in June 1801.

